# Selfish bacteria take up polysaccharides under deep ocean pressure: insights from in situ and ex situ measurements

**DOI:** 10.1093/ismeco/ycag138

**Published:** 2026-05-18

**Authors:** C Chad Lloyd, Laura Pareigis, John Paul Balmonte, Ronnie N Glud, Greta Reintjes, Carol Arnosti

**Affiliations:** Department of Earth, Marine and Environmental Sciences, University of North Carolina at Chapel Hill, Chapel Hill, NC 27599, United States; Microbial-Carbohydrate Interactions Group, Faculty of Chemistry/Biology, University of Bremen, 28359 Bremen, Germany; Department of Earth and Environmental Sciences, Lehigh University, Bethlehem, PA 18015, United States; Lehigh Oceans Research Center, Lehigh University, Bethlehem, PA 18015, United States; HADAL & Nordcee, Department of Biology, University of Southern Denmark, 5230 Odense M, Denmark; Danish Institute of Advanced Study (DIAS), University of Southern Denmark, 5230 Odense M, Denmark; Department of Ocean and Environmental Sciences, Tokyo University of Marine Science and Technology, 4-5-7 Konan, Minato-ku, Tokyo 108-8477, Japan; Microbial-Carbohydrate Interactions Group, Faculty of Chemistry/Biology, University of Bremen, 28359 Bremen, Germany; Department of Earth, Marine and Environmental Sciences, University of North Carolina at Chapel Hill, Chapel Hill, NC 27599, United States; Max Planck Institute for Marine Microbiology, 28359 Bremen, Germany

**Keywords:** hydrostatic pressure, carbon cycling, laminarin, enzymes, microbial metabolism, deep sea

## Abstract

Heterotrophic bacteria process a large fraction of the polysaccharides produced by phytoplankton in the surface ocean. Some of these polysaccharides are taken up by “selfish” bacteria, which bind, partially hydrolyze, and transport large fragments of polysaccharides into the cell with little loss of hydrolysis products. Recently, selfish bacteria have also been identified in deep ocean water. Whether these bacteria effectively capture polysaccharides under hydrostatic pressures typical of the deep ocean, however, remained an open question. Here, for the first time, we measured the extent of selfish uptake of laminarin—a common marine polysaccharide—in surface and deep waters, under pressures ranging from atmospheric up to ~50 MPa (equivalent to 5000 m depth). We incubated seawater from Japan, Denmark, and the western North Atlantic Ocean in pressure vessels and found that the extent of selfish uptake of laminarin varied somewhat by site but was insensitive to hydrostatic pressure. Deployment of an in situ incubator in the North Atlantic Ocean also showed evidence of selfish uptake in situ. These results suggest that selfish uptake can play a key role in polysaccharide degradation, especially in the deep ocean where other modes of organic matter degradation may be more inhibited by high hydrostatic pressure.

## Introduction

Heterotrophic bacteria use different mechanisms to access high molecular weight polysaccharides, major components of organic matter initially produced by phytoplankton in the surface ocean [1, 2]. Some bacteria (“external hydrolyzers”) use extracellular enzymes that are cell surface-attached or freely released to hydrolyze polysaccharides to sizes suitable for uptake [3]. These hydrolysis products may subsequently be available to non-enzyme producing (“scavenging”) bacteria that cannot or do not produce extracellular enzymes. Polysaccharides can also be taken up by “selfish” bacteria [4], which initially bind, partially hydrolyze, and transport large polysaccharides fragments into the periplasmic space, thereby retaining the hydrolysis products. This mechanism of polysaccharide uptake, first discovered in bacteria of the human gut [4], is also widespread in surface ocean waters [5–8]. Selfish bacteria are also active on particles [9] and in shallow marine sediments [10]. Selfish uptake in surface ocean waters has been found to vary with location and phytoplankton bloom stage [5–8, 11]; the varying relative balance of selfish uptake and external hydrolysis is likely related to polysaccharide abundance and structural complexity [12].

We previously hypothesized that selfish bacteria would be far more active and comparatively more numerous in surface waters, where they likely encounter a higher abundance of intact polysaccharides. However, recent investigations demonstrated that selfish bacteria are active throughout the water column of the ocean. In deep ocean waters, moreover, they take up substrates selfishly that are not hydrolyzed externally [13, 14]. These results suggest a mechanism for targeted removal of complex polysaccharides in the deep ocean, as well as implying a substantial export of polysaccharides to the deep, since rapidly sinking particles must provide sufficient substrate for the maintenance of a considerable bacterial population. These prior studies were carried out, however, with water from the deep ocean (depths of 3200–5580 m) that was maintained under in situ temperature, but at atmospheric pressure. Whether or not similar activities occur at hydrostatic pressures typical of the deep ocean remained unexplored. We therefore investigated the effects of elevated hydrostatic pressure on selfish bacterial uptake of polysaccharides in surface and deep ocean waters.

We carried out this investigation because high hydrostatic pressure affects many facets of bacterial physiology and therefore potentially also selfish uptake. For example, increased pressure compresses lipid bilayers, decreasing the distance between acyl chains, resulting in lateral shrinking and increased membrane thickness [15], in turn affecting ion channel permeability [16]. Specific membrane responses to elevated pressure cannot easily be generalized, however, since they depend on pressure effects on membrane polar headgroups and fatty acids [17], as well as on membrane-associated proteins [15]. Enzymes are also affected by elevated pressure, but no simple relationship has been found between the depth at which an organism is obtained and its enzyme activities under high pressure [18]. Moreover, differences in pressure responses can arise from subtle structural changes: the crystal structures of dihydrofolate reductase (essential for DNA synthesis) from the piezophile *Moritella profunda* and from *Escherichia coli* are virtually identical, yet the *M. profunda*-derived enzyme showed increased activity up to a pressure of 50 MPa, whereas activity of the *E. coli* dihydrofolate reductase decreased continuously under increasing pressure. These responses were attributed to differences in surface hydration and internal cavity volume [19]. Generalizing the effects of high hydrostatic pressure on enzymatic function is complicated by the fact that pressure affects enzyme cavity volumes as well as enzyme-associated waters of hydration. Moreover, the activation volume (ΔV) of a specific reaction, the volume difference between reactants and the transition state of a reaction, is the net outcome of many individual steps, each of which is affected individually by elevated pressure; the rate-limiting steps of these myriad reactions may also change with changes in hydrostatic pressure [18]. Therefore, unlike responses to temperature changes, physiological responses to changes in hydrostatic pressure are frequently non-linear and vary considerably among bacterial taxa [16].

In the ocean, bacteria on a particle sinking from the surface to the deep ocean would experience rapid increases in hydrostatic pressure [20–22]. In addition, deep-ocean bacteria must function in their environment despite the prevailing high hydrostatic pressure [23, 24]. Elevated hydrostatic pressure has been shown to affect bacterial growth rates (bacteria isolated from depths of 1400–5100 m; [25]), as well as bacterial protein production (pressures of 0.1–40 MPa, [26]; depths of 800–2000 m, [27]; depths of 1000–4000 m, [28]). The effects of elevated pressure vary considerably among individual bacteria (pressures up to 40 MPa, [29]) and the enzymes under consideration [19]. The specific substrate used also affects growth response under pressure, likely due to differences in metabolic pathways [30]. Measurements of extracellular enzyme activities, including exo-acting (terminal unit cleaving) leucine aminopeptidase (pressures up to 14 MPa; [31]) and several endo-acting (mid-chain cleaving) peptidases (pressures up to 100 MPa; [32]) have shown that these enzymes activities are affected by increased hydrostatic pressure, typically—but not always (pressures up to 10 900 m; [33])—showing a reduction in activity at high hydrostatic pressure. Some enzyme activities, however, particularly among sediment microbial communities, showed pressure insensitivity [32]. A recent investigation of polysaccharide hydrolase activities of surface-water bacterial communities found large decreases in activity with increasing hydrostatic pressure, although sediment-associated enzyme activities were less severely affected (pressures up to 40 MPa; [34]). Deep-sea communities, however, include organisms with different pressure responses [25, 32], such that some organisms from the deep function more effectively under elevated hydrostatic pressure than under atmospheric pressure [27, 35–37]. *Photobacterium profundum*, for example, induces specific polysaccharide hydrolase enzymes only under elevated pressures, shown to be activated at 28 MPa but not at 0.1 MPa [38].

Given these variable bacterial responses to high hydrostatic pressure and differences in bacterial community composition and function with location and depth in the ocean [39–41], we investigated the pressure responses of selfish bacteria that take up laminarin, a glucose-containing polysaccharide, in the surface and deep ocean. We collected water samples from surface (depths: 10–150 m) and deep waters (depths: 4000-5200 m) from three stations in the western North Atlantic Ocean and one station near the Japan Trench. We additionally collected surface water near the coast of Denmark. In all cases, surface water was incubated at atmospheric pressure and at pressure equivalent to the deep ocean; deep ocean water was incubated at atmospheric pressure and at in situ pressure. In addition, we developed and tested for the first time a deployable system to measure selfish uptake in situ. Combined, these investigations enabled us to investigate selfish uptake among different microbial communities and thereby to assess the potential importance of selfish uptake of polysaccharides as an aspect of carbon metabolism in the deep ocean.

## Materials and methods

### Stations and water collection

**Figure 1:**
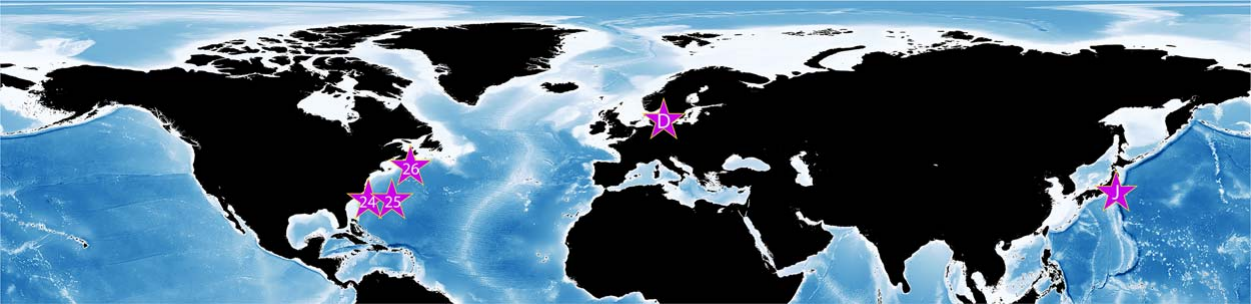
Location of sampling stations. J = Japan; D = Helsingør, Denmark. Western North Atlantic: the numbers 24, 25, and 26 refer to the three specific stations sampled (see text). Image modified from NASA (nasa.gov).

Our experiments were conducted at widely spaced locations in the western North Atlantic Ocean, the Pacific Ocean near the Japan Trench, and from a coastal site near Denmark (Fig. 1). In the western North Atlantic Ocean, water was collected aboard the R/V Atlantic Explorer in May 2024 at three stations: Station 24 (near the Gulf Stream: 34°59.16′N; 73°05.36′W; depth 4200 m), Station 25 (in the relatively oligotrophic Sargasso Sea: 34°59.81′N; 68°15.37′W; depth 5200 m), and Station 26 (in cooler, fresher waters off the continental shelf of Newfoundland, at 42°10.67′N; 60°03.27′W; depth 4200 m). At each station, water was sampled from the deep chlorophyll maximum (DCM) and from the bottom (Table 1), using a rosette (12 l Niskin bottles) with CTD (conductivity-temperature-depth) sensors. Water was transferred to 20 l carboys that were acid-washed and rinsed three times with reverse-osmosis water prior to sample collection. The in situ syringe system (see below) was deployed in bottom waters at Stations 24–26, and at a depth of 2000 m at Station 26.

**Table 1:** Water characteristics from the stations, as recorded via CTD (conductivity-temperature-depth sensors; chl *a* fluorescence values are also converted from the CTD sensors).

Location	Station	Depth	Depth (m)	Temperature (°C)	Salinity	Chlorophyll-*a* (mg m ^−3^ )
Denmark	–	20 m	20	12.9	32.5	0.15
Japan	7	Surface	10	26.6	34.5	–
		Bottom water	5000	1.5	34.7	–
North Atlantic Ocean	24	Bottom water	4000	2.2	34.9	0.03
	25	DCM	29	21.8	36.6	1.36
		Bottom water	5200	2.9	34.9	0.03
	26	DCM	38	5.8	33.0	3.45
		Bottom water	4200	2.2	34.9	0.03

DCM corresponds to the deep chlorophyll maximum, the depth of which varied by station.

In Japan, water was collected (October 2023) near the Japan trench (34°01.26′N; 141°86.06′W) at a depth of 10 m (surface water) and 5000 m (deep ocean) via CTD rosette with Niskin bottles aboard the R/V Umitaka Maru. After collection, water was transported back to the Japan Agency for Marine-Earth Science and Technology where experiments were conducted in the cold room at 4°C.

In Denmark, water was collected aboard the R/V Ophelia in September 2023 near Helsingør (55°57.90′N, 12°40.66′E), at 20 m depth (total water column depth: 25 m) with a Niskin rosette outfitted with a CTD.

The water was stored at 4°C, transported to the University of Southern Denmark, and experiments were initiated immediately in the cold room. Table 1 shows the chemical and physical characteristics of water from all stations.

### Experimental setup to measure selfish uptake of laminarin

Laminarin is a highly abundant polysaccharide in the ocean, with a net annual production of 5–15 billion metric tons [42]. Laminarin hydrolysis is widely measurable throughout the water column and sediments (e.g. [3, 43, 44]); it is also selfishly taken up by a broad range of bacteria [5–8, 11]. We therefore focused on selfish uptake of laminarin in this study. Fluorescently labeled (FLA)-laminarin was synthesized and characterized after [45]. At each station and depth, triplicate 50 ml Falcon tubes were used to set up incubations with FLA-laminarin at a final concentration of 3.5 μM monomer equivalent. This concentration can be used to detect hydrolytic activity but is sufficiently low to avoid enriching bacteria in bottom water, as seen in previous incubations showing unchanged cell numbers over seven days in bottom water from the western North Atlantic [13]. As a killed control, one 50 ml Falcon tube was filled with autoclaved seawater from each station. After the autoclaved water cooled, laminarin was added. After FLA-laminarin addition, the content of each Falcon tube was transferred to eight 6-mL exetainer vials (Labco) that were closed to exclude air. The water was subdivided for incubation because 6 ml exetainer vials are the largest size that fit into the pressure vessels and can withstand the elevated pressures used for experiments.

Samples were pressurized following [34]. In brief, exetainer vials were placed into stainless steel pressure vessels; water was added to the pressure vessel to exclude air [46]; the pressure vessels were then pressurized by hand pumps (to 40 MPa for Denmark; bottom water pressure equivalent for Japan and the North Atlantic Ocean). All samples were incubated at 4°C in the dark. Sample preparation and pressurization took ~15 min from substrate addition to pressurization. A comparable set of samples was incubated in exetainer vials at atmospheric pressure in the dark. After completing the experimental setup, an initial timepoint (*t*_0_) was collected (see below for details).

At each timepoint (Table 2), pressure vessels were depressurized and four 6-ml exetainer vials were combined and fixed with a concentration of 1% formaldehyde at room temperature (ca. 21°C) for 1 h, then filtered through either 25-mm diameter polycarbonate filters (0.2 μm pore size; Millipore; Japan and North Atlantic Ocean samples) or 47-mm diameter polycarbonate filters (0.2 μm pore size; Millipore; Denmark samples) and stored at −80°C. The combined four 6-ml exetainer vials therefore represent one replicate in the data shown below. In all, 12 live vials and 4 killed control vials were depressurized at each time point to obtain three live replicates and one killed-control replicate.

**Table 2:** Time course of sampling for selfish bacteria incubations.

Location	Station	Depth	*t* _ 0_	*t* _ 1_	*t* _ 2_
Denmark	–	20 m	~1 h	5 days	12 days
Japan	7	Surface	~1 h	24 h	48 h
		Bottom water	~1 h	24 h	48 h
North Atlantic Ocean	24	Bottom water	~20 min	44 h	–
	25	DCM	~20 min	45 h	–
		Bottom water	~20 min	48 h	–
	26	DCM	~20 min	5 days	–
		Bottom water	~20 min	5 days	–

For *t*_0_, these times reflect the time required for depressurizing and fixing samples that were subsequently filtered for total cell counts and selfish bacteria analysis. Note that multiple people worked on samples in the North Atlantic Ocean, resulting in the initial timepoint (*t*_0_) being processed more rapidly.

Incubations were carried out in this manner to ensure sufficient volume for bacterial cell counts and to optimally pack pressure-resistant vials into the 400 ml (internal volume) pressurization chambers. The unpressurized samples were treated in the same manner. In Denmark, timepoints were taken after 5-day and 12-day incubation. For Japan, timepoints were taken after 24 h and 48 h incubation. For the western North Atlantic Ocean samples, timepoints were taken after 48 h incubation for Stations 24 and 25, and after 5-day incubation at Station 26. The variation in timepoints allows us to examine selfish uptake after different incubation times. Varying experimental demands for pressure vessels—as well as uncertainty about when and whether pressure effects would be observable for selfish uptake—led us to use a variety of incubation times.

### In situ syringe system incubations

To assess the extent of selfish uptake at in situ temperature and pressure, a previously developed spring-loaded syringe sampler [47] was adapted to carry out in situ incubations. The modified sampler has 3 racks of seven 50 ml glass syringes, which were mounted on a 1.5 m tall octagonal stainless-steel frame (Fig. S1). The syringes are programmed to draw in water at pre-selected incubation timepoints. In this case, we programmed the syringe system to draw in water at bottom depths, for the incubation syringes (24 h at bottom depths) and for the upcast syringes, which were programmed to draw in water ca. 5 min prior to retrieval (see below).

To set up the system, the syringes were initially rinsed with 70% ethanol and then rinsed with MilliQ water at least three times prior to use. Note that the syringes could not be acid washed, due to potential corrosion of the metal fittings. FLA-laminarin was then pre-loaded into tubing that was affixed to the 50 ml glass syringes. These pre-loaded syringes were stored in the refrigerator for up to 2 h prior to syringe system deployment. Three syringes pre-loaded with autoclaved seawater served as blanks. Ten minutes before deployment, the syringes were secured in the rack on the syringe system (Fig. S1). The sampler was lowered by a winch to the desired water depth. At the target depth, four syringes drew in ambient water that mixed with the pre-loaded substrate. The sampler remained at the same depth for a period of 24 h, suspended on the winch wire. Shortly before recovery of the module (ca. 5 min prior to the start of the syringe system retrieval), two syringes were activated to draw in seawater in order to determine the extent of selfish uptake on the upcast (the “upcast” samples) until onboard fixation. During the upcast, therefore, the syringes were full of water that had been drawn in at depth. Note that the time to retrieve the syringe system depended on the deployment depth, but was between 54 min (for the 2000 m deployment) and 154 min (for the 5200 m deployment; Table 3). Once the syringe system was on deck, the syringes were removed and samples were processed as described above for analysis of selfish bacteria.

**Table 3:** Times for deployment, retrieval, incubation, and sampling for the in situ syringe system deployments.

Station	Depth (m)	Deployment time (UTC)	Trigger syringe *t* _1_ (UTC)	Trigger syringe upcast (UTC)	Time on deck (UTC)	Subsample time (UTC)
24	4200	2352	0235 + 1	0235 + 2	0425 + 2	0455 + 2
25	5200	1746	2016	2016 + 1	2250 + 1	2320 + 1
26	4200	0206	0500	0500 + 1	0650 + 1	0720 + 1
	2000	1426	1540	1540 + 1	1634 + 1	1704 + 1

We tested the reproducibility of FLA-laminarin mixing in the syringe volume at depth by collecting the filtrate from the fixed samples (see above) and quantifying the fluorescence signal via chromatography and fluorescence detection. The area of the resulting chromatograms was calculated and compared among syringes for single deployments, as well as between deployments. We then calculated the percent relative standard deviation from these values.

### Total cell counts and laminarin-stained cells

Total bacterial abundance and selfish bacterial abundance were quantified following [5, 48]. In brief, after fixation and filtration as described above, the DNA of filtered cells were simultaneously counterstained and mounted onto slides using a 1 ng/μl working solution of 4′,6-diamidin-2-phenylindol (DAPI) mixed with Citifluor/VectaShield (4:1). Automatic images for total cell counts and selfish cell counts were acquired with an automated imaging system (Zeiss AxioImager.Z2 microscopic stand, Carl Zeiss) that used LEDs with a 63× magnification oil immersion plan apochromatic objective with a numerical aperture of 1.4 (Carl Zeiss). Typically, a minimum of 10 images is used per sample for analysis; we set the software to obtain 37 images for surface samples and 63 images for deep samples. Final cell counts were made using ACMETOOL 3 (http://www.tchnobiology.ch; Max Planck Institute for Marine Microbiology, Bremen). Quantification of DAPI-stained cells is reported as total cell abundance. Cells that had a minimum overlap of 35% of DAPI signal and fluorescently-labeled laminarin signal (signal: background >1.5) are reported as selfish bacteria.

### Super-resolution structured illumination microscopy

Microscopic images were obtained using a Zeiss ELYRA PS.1 (Carl Zeiss) with 561, 488, and 405 nm lasers and BP 573–613, BP 502–538, and BP420–480 + LP 750 optical filters, following procedures described in detail in [5].

### Reproducibility of syringe system substrate injections

To determine reproducibility of laminarin concentrations between syringes on the syringe system, we injected a small amount of the filtrate from our selfish bacteria incubations onto a gel permeation chromatography system with a fluorescence detector [45]. From the area of the chromatograms, we calculated the percent relative standard deviation among syringes to compare the laminarin concentrations in situ among syringes when filled with seawater. This value for each deployment ranged from 6.2% to 15.5% (Table S1), relatively low for biological replicates.

### Statistical analyses

Data were analyzed in R version 4.3.0 [49]. The percentage abundance of FLAPS-stained cells was converted to a proportion (0–1) to ensure compatibility with the beta distribution and logit link. A zero-inflated beta regression model was fitted using the ‘glmmTMB’ package [50], chosen to account for excess zeros and random effects (i.e. cruise). The model included fixed effects of pressure condition (in situ vs. adjusted) and depth (surface vs. deep), their interaction, and cruise as a random intercept. The analysis included 120 observations (surface *n* = 54, deep *n* = 66) across 4 cruises. Each observation is the average of a minimum of 15 fields of view (microscope images). Model fit was assessed using AIC and likelihood ratio tests. Post hoc comparisons were conducted with the ‘emmeans’ package [51].

In addition, *t*-tests (parametric, one-tailed, paired) were performed to compare incubations at individual sites and depths within and between timepoints for the pressurized and unpressurized samples (i.e. pressurized *t*_1_ vs. *t*_0_ and unpressurized *t*_1_ vs. *t*_0_): ▪ = *P* < .10; ^*^ = *P* < .05; ^**^ = *P* < .01; ^***^ = *P* < .001.

## Results

### Selfish uptake under elevated pressure

**Figure 2:**
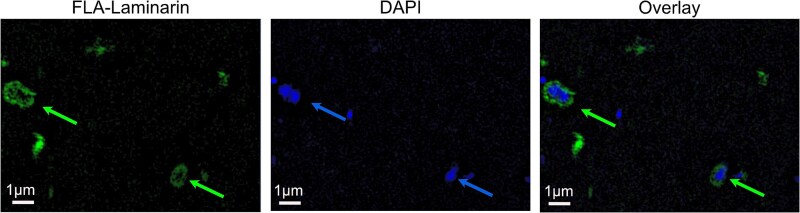
Super resolution-structured illumination microscopy image of fluorescently labeled laminarin uptake by bacterial cells from Danish waters incubated for 5 days under 40 MPa hydrostatic pressure (equivalent to 4000 m depth). In this incubation, both the percentage and the number of selfish bacteria increased with incubation time, demonstrating selfish uptake under high hydrostatic pressure. Cells (shown by arrows) were stained with DAPI (center panel) and show uptake of fluorescently labeled laminarin (left panel) in a halo-link fashion, indicating uptake into the periplasm. Right panel shows overlay of the left and center images.

**Figure 3:**
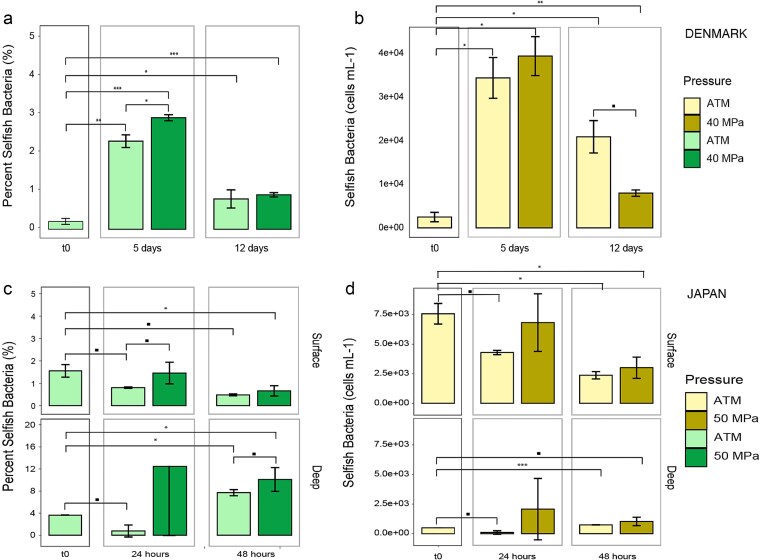
(a) Percent selfish bacteria and (b) selfish bacterial abundance in Danish coastal waters under atmospheric pressure and 40 MPa (equivalent to 4000 m depth) at 0 h, 5 days, and 12 days. Bars represent the average of triplicate incubations while error bars represent the standard deviation of these measurements. (c) Percent selfish bacteria and (d) selfish bacterial abundance from surface and deep ocean waters (5000 m) near the Japan trench under atmospheric pressure and 50 MPa (equivalent to 5000 m depth) at 0, 24, and 48 h. Bars show the average of triplicate incubations while error bars represent the standard deviation of these measurements. *t*-tests (parametric, one-tailed, paired) were performed to compare incubations within and between timepoints for the pressurized and unpressurized samples (i.e. pressurized *t*_1_ vs. *t*_0_ and unpressurized *t*_1_ vs. *t*_0_): ▪ = *P* < .10; ^*^ = *P* < .05; ^**^ = *P* < .01; ^***^ = *P* < .001.

**Figure 4:**
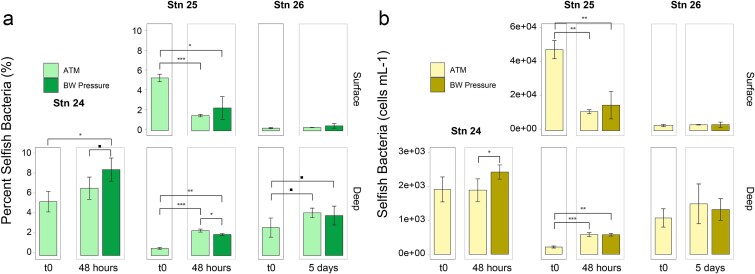
(a) Percent selfish bacteria and (b) selfish bacterial abundance from surface and deep ocean waters at three stations in the North Atlantic Ocean. Samples were incubated under atmospheric pressure or pressures equivalent to bottom water pressure at each station. Bars show the average of triplicate incubations while error bars represent the standard deviation of these measurements. Station 24 was in the Gulf Stream, Station 25 was in the relatively oligotrophic subtropical gyre, and Station 26 was in cool, relatively fresh, productive waters off the continental slope of Newfoundland (Fig. 1). *t*-tests (parametric, one-tailed, paired) were performed to compare incubations within and between timepoints for the pressurized and unpressurized samples (i.e. pressurized *t*_1_ vs. *t*_0_ and unpressurized *t*_1_ vs. *t*_0_): ▪ = *P* < .10; ^*^ = *P* < .05; ^**^ = *P* < .01; ^***^ = *P* < .001.

**Figure 5:**
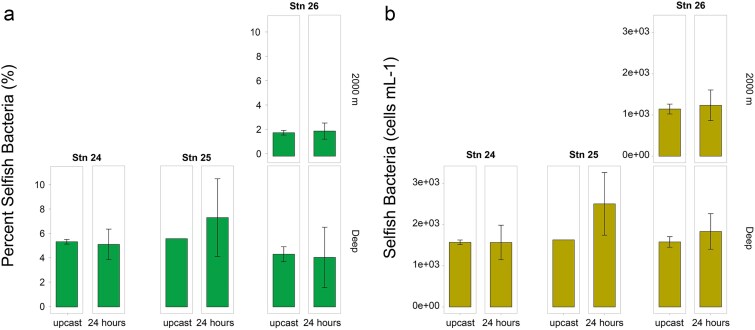
(a) Percent selfish bacteria and (b) selfish bacterial abundance of samples collected from in situ syringe system deployments at either 2000 m or bottom water (deep ocean) in the western North Atlantic Ocean. Bars show the average while error bars represent the standard deviation of these incubations. “Upcast” denotes samples collected from syringes that were triggered 5 min prior to initiating the retrieval of the syringe system from depth. The other samples were incubated 24 h at in situ temperature and pressure prior to syringe system retrieval. Station 24 was in the Gulf Stream, Station 25 was in the relatively oligotrophic subtropical gyre, and Station 26 was in cool, relatively fresh, productive waters off the continental slope of Newfoundland.

Across all stations and depths, microscopic analysis of samples showing DAPI staining of DNA (blue) and the halo-like staining from fluorescently-labeled laminarin (green; Fig. 2) provides strong evidence that laminarin was taken up selfishly by bacteria (Figs 3–5). The percentage of cells showing this pattern, which—in accordance with previous investigations we interpret as selfish uptake [5–8, 10, 11, 13, 14, 48]—ranged from 1%–12%, depending on location and depth (Figs 3–5). These observations hold across all stations and depths, and incubation times ranging from 24 h to 12 days. Killed control samples did not show any similar patterns (Fig. S2).

The extent of selfish uptake in surface waters of samples that were incubated below their in situ temperatures—surface waters from Station 25, from Denmark, and from Japan—could be underestimated, since bacterial metabolism is affected by temperature. However, the extent of such an effect is unknown, since temperature effects on selfish uptake have yet to be investigated. In any case, selfish uptake under atmospheric and at elevated hydrospheric pressure was also observed in surface waters of Station 26, as well as bottom water incubations from Japan, and Stations 24, 25, and 26, where the in situ and incubation temperatures were very similar (Table 1; Figs 3c, d, and 4). Our rationale for this experimental design, given logistical constraints, was to focus specifically on pressure effects on selfish uptake. For surface waters, using the same experimental temperature as for deep water investigations also permits an estimate of the extent to which surface organisms that may sink to the deep ocean—attached to sinking particles, for example—might contribute to selfish uptake in the deep.

### Location- and depth-related differences in extent of selfish uptake of laminarin

In Danish coastal waters, selfish uptake of laminarin increased under atmospheric pressure between *t*_0_ and 5 days, but increased even more under a pressure of 40 MPa, considering selfish bacteria as a fraction of total cell counts (Fig. 3a; Table S3). Since total cell count did not change substantially between *t*_0_ and day 5 (Fig. S3), an increasing fraction of a relatively constant number of bacteria selfishly took up laminarin by day 5. After 12 days, total bacterial counts increased substantially under atmospheric pressure but declined slightly when incubated at 40 MPa (Fig. S3). As a fraction of total cells, the 12-day incubations also showed more selfish uptake compared to the *t*_0_ samples, but the extent of selfish uptake was less than in the 5-day samples; fewer cells overall, as well as a lower fraction of the community, showed the presence of FLA-laminarin in the periplasmic space.

In waters collected near the Japan Trench, the percentage of selfish bacteria was considerably higher in deep waters than in surface waters and was generally higher under 50 MPa incubation pressure than under atmospheric pressure, especially for the deep waters (Fig. 3c; Table S2), although the total number of selfish bacteria was smaller in deep than in surface waters (Fig. 3d; Table S3). Total bacterial abundance in both surface and deep waters was generally constant over the 48 h incubation (Fig. S4).

In the western North Atlantic Ocean, the percentage of selfish bacteria was generally comparable (and at Station 24, was higher) under elevated pressure compared to atmospheric pressure (Fig. 4; Table S4). Especially in bottom waters, the percentage of selfish bacteria was higher at 48 h compared to the *t*_0_ sample. In surface waters, the percentage at 48 h relative to *t*_0_ was lower at Station 25, and very similar at Station 26. Selfish bacterial abundance in bottom water increased after 48 h incubation (Stations 24 and 25) as well as 5-day incubation (Station 26) compared to *t*_0_ (Fig. 4), even though total cell abundance decreased slightly over these same time intervals (Fig. S5). Total cell numbers were very similar in pressurized and unpressurized bottom water samples (Fig. S5). In surface waters, total cell abundance was lower—especially for incubations under elevated hydrostatic pressure—after 48 h at Station 25 and 5 days at Station 26 (Fig. S5). Selfish bacterial abundance was also considerably lower in surface waters at Station 25 after 48 h incubation, whereas at Station 26, selfish bacterial abundance was comparable to *t*_0_ after 5-day incubation (Fig. 4b).

Overall, the percentage of selfish bacteria varied among stations, but did not exceed 12% of the total cells. Selfish bacteria off the coast of Denmark made up ca. 1%–3% of all bacteria (Fig. 3a), whereas selfish bacteria in waters collected near the Japan Trench were ca. 1%–2% of total surface ocean bacteria, and 2%–12% of the total population at 5000 m (Fig. 3c). In the western North Atlantic Ocean, selfish bacteria ranged from 1%–10% of total bacterial cells (Fig. 4). When considered by station (rather than for the entire suite of measurements), the percentage of selfish bacteria was higher at depth (Fig. S6), regardless of whether the samples were pressurized or not. However, all sample preparation and incubations were carried out in cold rooms at 4°C. This temperature is similar to the temperature of bottom water in Japan and at Stations 24–26, and to DCM water at Station 26, but considerably colder than surface waters collected in Japan, Denmark, and at Station 25 (Table 1). Therefore, lower incubation temperature may have led to lower bacterial activities at some surface locations.

### Selfish uptake in the in situ syringe system incubations

The in situ syringe system (Fig. S1) was used to measure selfish uptake of polysaccharides directly at depth. Due to logistic constraints, we could only deploy the system for 24 h periods. These deployments were at bottom water depths for Stations 24–26 (Table 3), with an additional deployment at 2000 m for Station 26. Selfish uptake of laminarin occurred at all stations and depths, but the extent of selfish uptake was very similar for the upcast samples and for the samples incubated for 24 h prior to the upcast (Fig. 5). The percentage of cells taking up laminarin was generally higher in bottom water depths (4–8%) compared to the samples incubated at 2000 m (~2%; Fig. 5) for Station 26, where incubations were carried out at both depths. The total bacterial abundance was similar for bottom water at all stations, both in the upcast and the 24 h incubations (Fig. S7; Table S6), suggesting limited impact of recovery for both the total cell numbers and the relative proportion of selfish bacteria.

### Statistical analysis of selfish uptake across all sites and depths

Analyzed across all sites and depths, zero-inflation beta regression revealed no significant effect of pressure condition (adjusted vs. in situ; *P* = .53), depth (surface vs. deep; *P* = .96) or their interactions (*P* = .33) on the abundance of selfish cells, with excess zeros in the data (*P* < .001*)* indicating frequent absence of selfish activity across the sampling sites. The abundance of selfish cells thus was not significantly affected by pressure condition, depth, or their interaction (Table 4).

**Table 4:** Statistical analysis of selfish uptake across all depths and stations.

Depth	Mean proportion	Standard error (SE)	95% confidence interval (CI)	*N* samples
Surface	0.000169	2.54 × 10^−5^	0.000126–0.000227	*n* = 54
Deep	0.000139	2.05 × 10^−5^	0.000104–0.000185	*n* = 66

## Discussion

The sinking flux of organic matter in the ocean supplies mesopelagic depths and the deep benthos with food, affects nutrient redistribution in the ocean, and controls the atmospheric balance of oxygen and CO_2_ over geologic timescales [52–54]. The fate of this material depends in large part on the capabilities of heterotrophic microbial communities that process organic matter as it sinks to greater depths. The functioning of these microbial communities in turn is affected by environmental conditions, particularly in situ temperature and pressure [55–57]. Efforts to quantify microbially catalyzed degradation processes therefore should also include assessing microbial processes under the pressure conditions typical of the deep sea (e.g. [29, 32, 58]). Here, we present the first investigation of selfish uptake of polysaccharides under elevated hydrostatic pressure.

**Figure 6:**
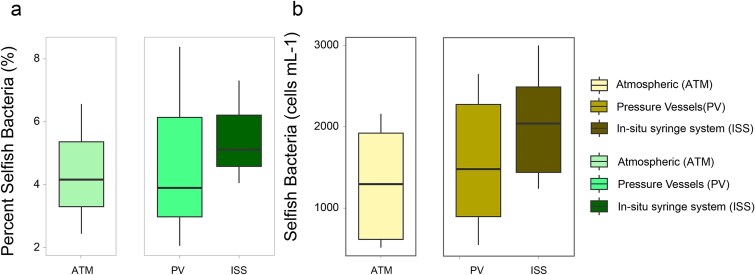
(a) Percentage of selfish bacteria and (b) selfish bacterial abundance in bottom water samples that were incubated under atmospheric conditions, re-pressurized to bottom water depths, or kept under in situ pressure with the in situ syringe system. There is no significant difference in selfish uptake between samples from the in situ syringe system, those incubated under atmospheric pressure, and those incubated under elevated hydrostatic pressure (ANOVA, *P* = .63). The box represents the upper and lower quartiles of the dataset while the line represents the median; whiskers represent the standard deviation.

Selfish uptake of laminarin in our repressurization experiments was largely insensitive to pressure: selfish uptake under pressure was comparable to or higher than selfish uptake at atmospheric pressure (Figs 3–5). Moreover, the observation that the overall percentage and number of selfish cells was similar under atmospheric as well as elevated pressure (Fig. 6) suggests that the pressurization/depressurization process itself did not measurably affect cell counts or the FLA-laminarin content of cells. However, the samples from the in situ syringe system that were incubated in the deep ocean present a mixed picture: only in Station 25 bottom water was the number of selfish cells (Fig. 5b) higher in the samples incubated for 24 h than in the samples triggered immediately prior to the upcast, indicating selfish uptake in situ. In Station 26 incubations at 2000 m and in bottom water, although the average selfish bacterial abundance was greater in the 24 h samples than in the upcast samples, these differences were not statistically significant (*t-*test, non-parametric, two-tailed distribution, heteroscedastic, *P* = .68, and *P* = .55, respectively). At Station 24, there was no difference in the extent of selfish uptake in the 24 h and the upcast samples (Fig. 5). In this case, it is possible that the activity in both sets of incubations occurred during the upcast and depressurization, since selfish uptake can occur on short timescales [5, 13]. We hypothesize, however, that the upcast activity—as well as the activity measured in the 24 h incubations—likely represents the rapid response of the fraction of the community that was primed to carry out selfish uptake in the deep ocean. In support of this perspective, we found that the percentage of bacteria showing selfish uptake of laminarin with the in situ syringe system was similar to the percentage of the bacterial community taking up laminarin in the pressure vessel incubations of bottom water (Fig. 6). This percentage of the community—ca. 4–5% in bottom waters—is also comparable to the fraction of the community that carried out selfish uptake of laminarin at *t*_0_ and 24 h timepoints in the previous investigation of selfish uptake at three stations in deep waters of the western North Atlantic Ocean [13]. The possibility that all the selfish uptake at Stations 24 and 26 occurred during recovery of the syringe system when samples were being de-pressurized should also be considered. However, the pressure vessel incubations of bottom water from Stations 24–26 (Fig. 5), the 5-day incubations with coastal waters from Denmark (Fig. 3a) and the 48 h incubations of bottom water from a depth of 5000 m off Japan (Fig. 3b) all show an increase in selfish uptake compared to the *t*_0_ samples (Tables S4 and S5), an observation that would not be consistent with selfish uptake occurring only during the depressurization phase of sample handling. The syringe system, which was adapted to investigate in situ selfish uptake in the ocean, was deployed for the longest time interval available during this field project. Going forward, a longer phase of deep ocean incubation would be desirable. In addition, further development of the in situ incubator system to carry out filtration and fixation at depth would provide the means to distinguish more clearly between in situ activity and activity during the upcast.

Selfish uptake of laminarin varied by station as well as timepoint (Figs 3 and 4), observations consistent with previous observations in surface [6] and deep ocean waters [13]. Station-related differences, especially as seen in the surface waters of Stations 25 and 26, could be driven in part by higher primary productivity (chl *a*; Table 1) at Station 26, since an increase in primary productivity during a spring bloom can lead to an increase in external hydrolysis concurrent with a decrease in selfish uptake [8]. Lower absolute and relative counts of selfish bacteria at later timepoints (e.g. Denmark 12 d vs. 5 d; Station 25 surface, 48 h vs. t₀; Figs 3, 4) may reflect loss of the FLA tag from the periplasm, as observed in *Christiangramia forsetii* [5]. However, due to the use of pressure vessels, we could not subsample replicate incubations over time (as is done in experiments not using pressure vessels) because opening the vessels required immediate filtration of all vials. This limited our time course to discrete, non-overlapping measurements, preventing assessment of dynamic changes. We therefore do not know if selfish uptake might have been higher had we measured at different timepoints at any given station. Future studies with more pressure vessels and extended ship time could enable longer time-course measurements. However, our primary goals were to assess the effect of hydrostatic pressure on selfish uptake relative to atmospheric controls and to demonstrate the feasibility of measuring this process in situ. The precise dynamics of uptake over time—such as whether our timepoints captured peak uptake—were not the central focus.

Station-related differences in selfish uptake may also be due to incubation temperature, since temperature can affect enzyme kinetics, diffusion rates, and protein interactions [59]. All surface/DCM water incubations—including the surface/DCM waters from coastal Denmark, off the Japan Trench, and at Stations 25 and 26—were incubated at 4°C, a temperature similar to the in situ temperature only in Station 26 surface waters (Table 1). The comparatively low fraction of selfish uptake in surface/DCM waters that we measured (Figs 3 and 4) relative to previous investigations (e.g. [13]) may be due to this lower incubation temperature, although a range of selfish uptake of laminarin—including similarly low percentages in surface waters at some locations—has been reported [6]. The observation that selfish uptake in DCM waters of Station 26 was notably low despite the similarity of in situ and incubation temperatures, however, suggests that at least at this station, the abundance of available organic matter and potentially higher external hydrolysis may have been a major control on selfish uptake of laminarin.

Our assessment of selfish uptake is made at a community level, comparing the number of cells showing selfish uptake of laminarin at a given timepoint. Because individual bacteria react differently to changes in hydrostatic pressure [29], selfish uptake under pressure—especially for longer incubations—may be associated with different microorganisms. In surface water communities, particularly those associated with diatom aggregates, initially minor members have been found to become more important under increased hydrostatic pressure [55]. In Danish coastal waters and Station 26 surface waters that were incubated for 5 days, such a change in community composition may have favored bacteria that could selfishly take up laminarin. Especially for the shorter (24 h) incubations that provided less time for changes in community composition, however, favorable pressure-related effects on enzyme cavities and hydration (e.g. [19]) could contribute to the observed resilience of selfish uptake. The polysaccharide used in these experiments may also have played a role: laminarin is abundant in the ocean [42, 60] and is taken up selfishly by a wide range of bacteria [6, 11, 14] that may exhibit differing pressure sensitivities. In the future, measuring selfish uptake of other polysaccharides that are taken up by a narrower range of bacteria could provide the means to examine pressure effects on a more focused range of organisms.

The observation that the extent of selfish uptake in surface ocean waters, as measured under our experimental conditions, is not affected by elevated hydrostatic pressure (Figs 3–5) contrasts strikingly with the results from experiments investigating effects of hydrostatic pressure on external (extracellular) hydrolysis of various polysaccharides, including laminarin. Bacterial communities in the same Danish coastal waters, collected, incubated, and processed simultaneously with the selfish uptake experiments described here, showed greatly reduced extracellular enzyme activities under high hydrostatic pressure [34]. Several enzyme activities measured at atmospheric pressure were not measurable at a pressure of 20 MPa (equivalent to a depth of 2000 m in the ocean), and none of the enzyme activities tested were measurable at a pressure of 40 MPa (equivalent to a depth of 4000 m in the ocean). This observation suggests that selfish bacteria—along with other piezotolerant taxa—that are transported from the surface to the deep ocean may continue to process high molecular weight organic matter unimpeded by changes in hydrostatic pressure. Given the pressure sensitivity of external hydrolysis among surface water communities [34], and a typical decrease in the spectrum of enzyme activities measurable in deep ocean water incubated under atmospheric pressure (e.g. [43, 44]), selfish uptake may be a major route by which polysaccharides transported intact to the deep ocean [61, 62] are processed by heterotrophic communities. These particles—and polysaccharides—would constitute the substrates needed for survival in resource-limited environments [63]. Future investigations incorporating additional polysaccharides, community analyses, and in situ filtering and fixation of samples will help further reveal the role of individuals as well as communities in this important process.

## Supplementary Material

Selfish_Uptake_of_Laminarin_SI_revised_ycag138

## Data Availability

The data in this manuscript are available in the Biological and Chemical Oceanography Data Management Office at https://www.bco-dmo.org/dataset/963393. They can be accessed using the following DOI: 10.26008/1912/bco-dmo.963393.1.
