# Supplementary material for: Selfish bacteria take up polysaccharides under deep ocean pressure: insights from in situ and ex situ measurements
Source: ISME Commun. 2026 May 18;6(1):ycag138. doi: 10.1093/ismeco/ycag138 (PMC13286000; doi:10.1093/ismeco/ycag138)
Supplement: Selfish_Uptake_of_Laminarin_SI_revised_ycag138 [file selfish_uptake_of_laminarin_si_revised_ycag138.pdf]

Supplemental Tables and Figures

Table S1. Reproducibility FLA-laminarin concentrations in the syringe volume at depth, as measured via the fluorescence of the filtrate from fixed samples. The area of the chromatogram (fluorescence signal), the standard deviation of the area under the curve, and the percent relative standard deviation for each deployment were calculated.

Deployment	Depth (m)	Area Under Curve	Standard Deviation	Percent Relative Standard Deviation
SSD1	4000	79828	7681	9.6%
SSD2	5200	58820	8410	14.3%
SSD3	4200	67918	4203	6.2%
SSD4	2000	43858	6530	15.5%

Table S2. A comparison of the average percentage of selfish cells in pressurized and unpressurized incubations at each timepoint. The pressurized and unpressurized incubations at single timepoints were compared using T-tests (parametric, one-tailed, paired): ▪ = p<0.10; * = p<0.05; ** = p<0.01; *** = p<0.001. Note that the statistical notation is placed on the sample with the higher percentage of selfish cells. ATM = atmospheric pressure.

				Percent selfish cells (%)	
Location	Station	Depth	Pressure	t_1	t_2
Denmark	-	20 m	ATM	2.29	0.79
			40 MPa	2.90*	0.90
Japan	7	Surface	ATM	0.80	0.48
			50 MPa	1.46▪	0.66
		Bottom Water	ATM	0.77	7.71
			50 MPa	12.5	10.1▪
North Atlantic Ocean	24	Bottom Water	ATM	6.57	-
			42 MPa	8.38▪	-
	25	DCM	ATM	1.50	-
			52 MPa	2.25	-
		Bottom Water	ATM	2.44*	-
			52 MPa	2.06	-
	26	DCM	ATM	0.32	-
			42 MPa	0.48	-
		Bottom Water	ATM	4.16	-
			42 MPa	3.90	-

Table S3. A comparison of the average selfish cell abundance in pressurized and unpressurized (ATM, atmospheric pressure) incubations at each timepoint. The pressurized and unpressurized incubations at single timepoints were compared using T-tests (parametric, one-tailed, paired): ▪ = $p<0.10$; * = $p<0.05$; ** = $p<0.01$; *** = $p<0.001$. Note that the statistic notation is placed on the sample with the higher percentage of selfish cells. ATM = atmospheric pressure.

				Selfish cell abundance (cells mL^{-1})	
Location	Station	Depth	Pressure	t_1	t_2
Denmark	-	20 m	ATM	3.54E+04	2.15E+04▪
			40 MPa	4.06E+04	8.20E+03
Japan	7	Surface	ATM	4.29E+03	2.73E+03
			50 MPa	6.82E+03	3.01E+03
		Bottom Water	ATM	1.04E+02	7.46E+02
			50 MPa	3.11E+03	1.03E+03
North Atlantic Ocean	24	Bottom Water	ATM	1.89E+03	-
			42 MPa	2.42E+03*	-
	25	DCM	ATM	1.12E+04	-
			52 MPa	1.51E+04	-
		Bottom Water	ATM	5.78E+02	-
			52 MPa	5.75E+02	-
	26	DCM	ATM	3.46E+03	-
			42 MPa	3.51E+03	-
		Bottom Water	ATM	1.49E+03	-
			42 MPa	1.32E+03	-

Table S4. A comparison of incubations across timepoints, showing the average percentage of selfish cells for incubations in pressure vessels. T-tests (parametric, one-tailed, paired) were performed to compare incubations between timepoints for the pressurized and unpressurized samples (i.e., pressurized t_1 vs. t_0 and unpressurized t_1 vs. t_0): ▪ = $p<0.10$; * = $p<0.05$; ** = $p<0.01$; *** = $p<0.001$. ATM = atmospheric pressure.

				Percent selfish bacteria (%)		
Location	Station	Depth	Pressure	t_0	t_1	t_2
Denmark	-	20 m	ATM	0.20	2.29**	0.79*
			40 MPa		2.90***	0.90***
Japan	7	Surface	ATM	1.55	0.80▪	0.48▪
			50 MPa		1.46	0.66*
		Bottom Water	ATM	3.64	0.77▪	7.71*
			50 MPa		12.5	10.1*
North Atlantic Ocean	24	Bottom Water	ATM	5.25	6.57	-
			42 MPa		8.38*	-
	25	DCM	ATM	5.20	1.50***	-
			52 MPa		2.25*	-
		Bottom Water	ATM	0.70	2.44***	-
			52 MPa		2.06**	-
	26	DCM	ATM	0.27	0.32	-
			42 MPa		0.48	-
		Bottom Water	ATM	2.73	4.16▪	-
			42 MPa		3.90▪	-

Table S5. A comparison of incubations across timepoints, showing the average selfish bacterial abundance for pressure vessel incubations. T-tests (parametric, one-tailed, paired) were performed to compare the pressurized and unpressurized incubations between timepoints: ▪ = p<0.10; * = p<0.05; ** = p<0.01; *** = p<0.001. ATM = atmospheric pressure.

				Selfish cell abundance (cells/mL)		
Location	Station	Depth	Pressure	t_0	t_1	t_2
Denmark	-	20 m	ATM	2.55E+03	3.54E+04*	2.15E+04*
			40 MPa		4.06E+04*	8.20E+03**
Japan	7	Surface	ATM	7.57E+03	4.29E+03▪	2.73E+03*
			50 MPa		6.82E+03	3.01E+03*
		Bottom Water	ATM	4.98E+02	1.04E+02▪	7.46E+02***
			50 MPa		3.11E+03	1.03E+03▪
North Atlantic Ocean	24	Bottom Water	ATM	1.91E+03	1.89E+03	-
			42 MPa		2.42E+03	-
	25	DCM	ATM	4.77E+04	1.12E+04**	-
			52 MPa		1.51E+04**	-
		Bottom Water	ATM	2.13E+02	5.78E+02***	-
			52 MPa		5.75E+02**	-
	26	DCM	ATM	3.04E+03	3.46E+03	-
			42 MPa		3.51E+03	-
		Bottom Water	ATM	1.07E+03	1.49E+03	-
			42 MPa		1.32E+03	-

Table S6. The average selfish bacterial abundance and percentage of selfish cells in the in-situ syringe system incubations. T-tests (non-parametric, two-tailed distribution, heteroscedastic) were performed to compare the upcast and 24-hr incubations: ▪ = p<0.10; * = p<0.05; ** = p<0.01; *** = p<0.001. $^{\Delta}$ represents samples that could not be statistically compared due to a lack of replicates.

		Selfish bacteria (cells/mL)		Percent selfish bacteria (%)	
Station	Depth	Upcast	24 hrs	Upcast	24 hrs
24	Bottom Water	1.57E+03	1.57E+03	5.36	5.11
25	Bottom Water	1.63E+03$^{\Delta}$	2.51E+03$^{\Delta}$	5.58$^{\Delta}$	7.31$^{\Delta}$
26	2000 m	1.15E+03	1.24E+03	1.93	2.05
	Bottom Water	1.58E+03	1.84E+03	4.31	4.05

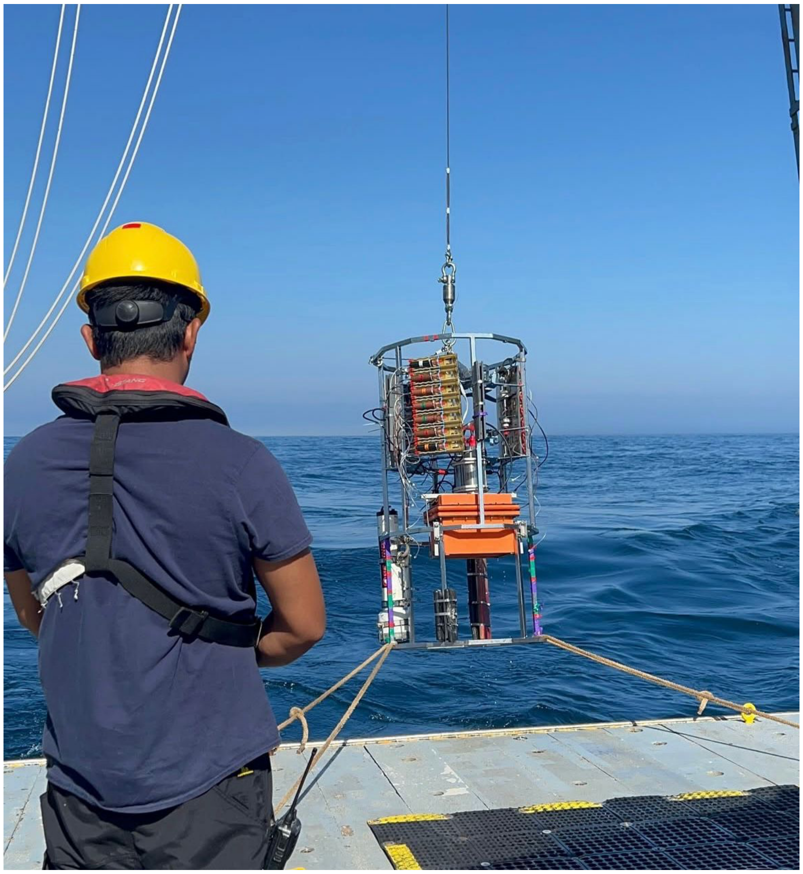

Figure S1: In situ syringe system being deployed (photo: D. Sathe).

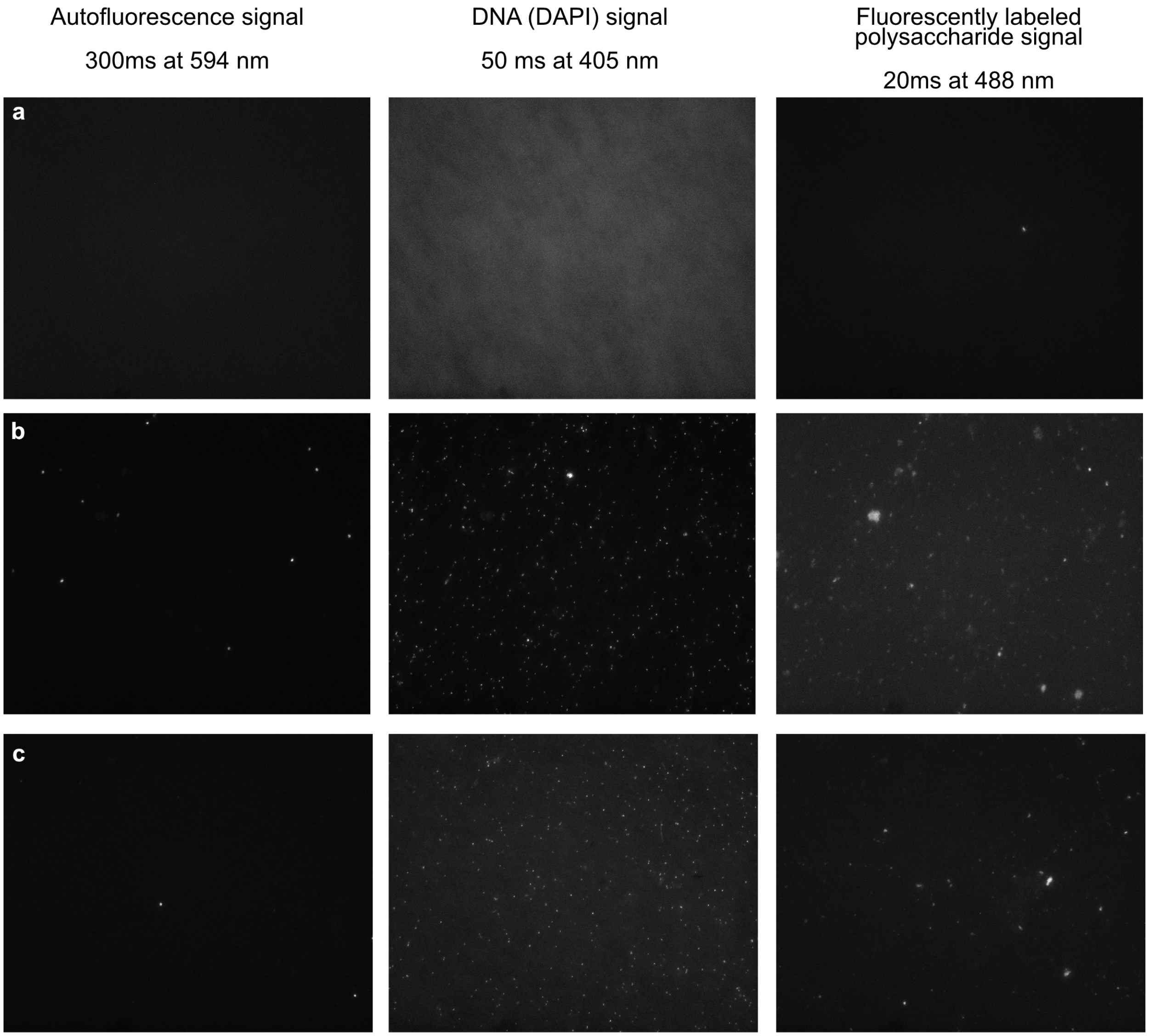

Fig. S2: Microscope images of **a**) killed controls sample and **b)** and **c**) two fluorescently labelled substrate incubation samples. Images are from the automated image acquisition system (see main text for details) showing example of autofluorescence at 594 nm wavelength with 300 ms exposure time, DNA signal stained by DAPI and acquired at 405 nm wavelength with 50 ms exposure time and fluorescently labeled substrate signal at 488 nm wavelength with 20 ms exposure time. Cells were only counted as positive for selfish uptake if there was a DAPI signal and an overlapping fluorescently labeled substrate signal but no autofluorescence signal. Images were taken using 63x magnification oil immersion plan apochromatic objective with a numerical aperture of 1.4 (Carl Zeiss).

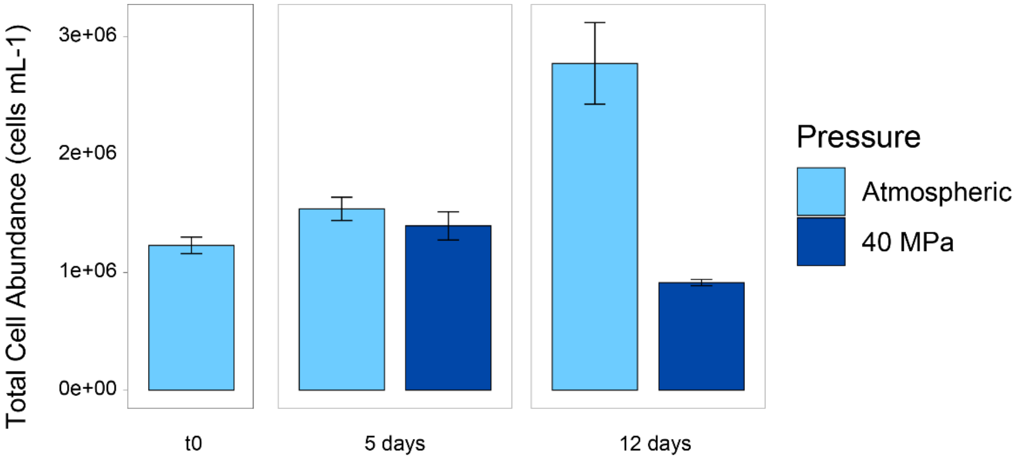

Figure S3: Total cell abundance in Danish coastal waters under atmospheric pressure and 40 MPa (equivalent to 4000 m depth) at different timepoints. Bars represent the average of triplicate incubations while error bars represent the standard deviation of these incubations.

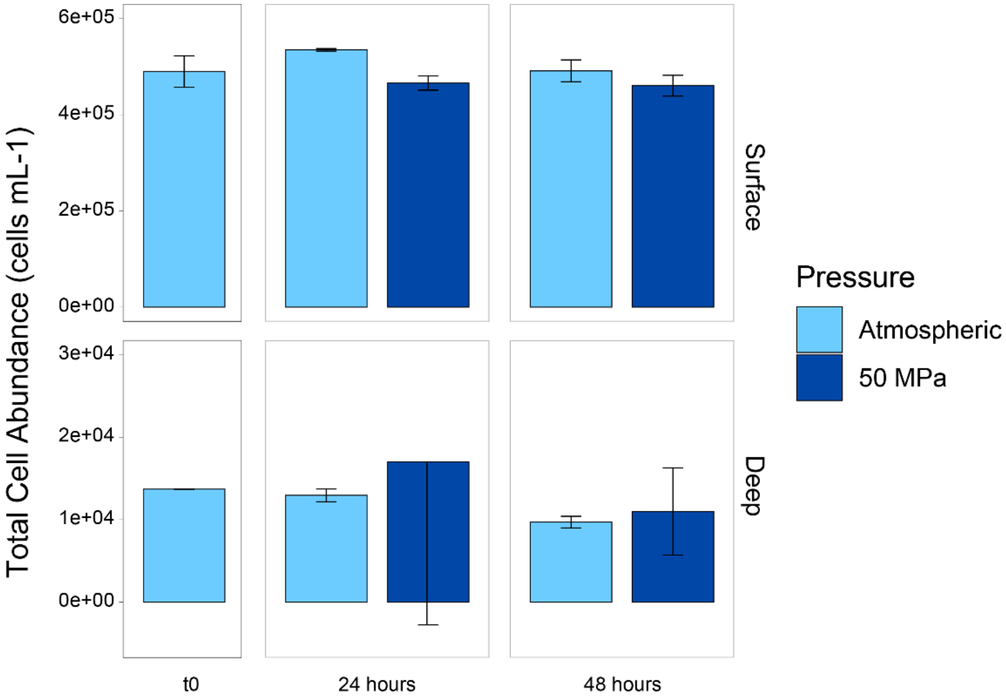

Figure S4. Total cell abundance from surface and deep ocean waters near the Japan Trench under atmospheric pressure and 50 MPa (equivalent to 5000 m depth) at different timepoints. Bars show the average of triplicate incubations while error bars represent the standard deviation of these incubations.

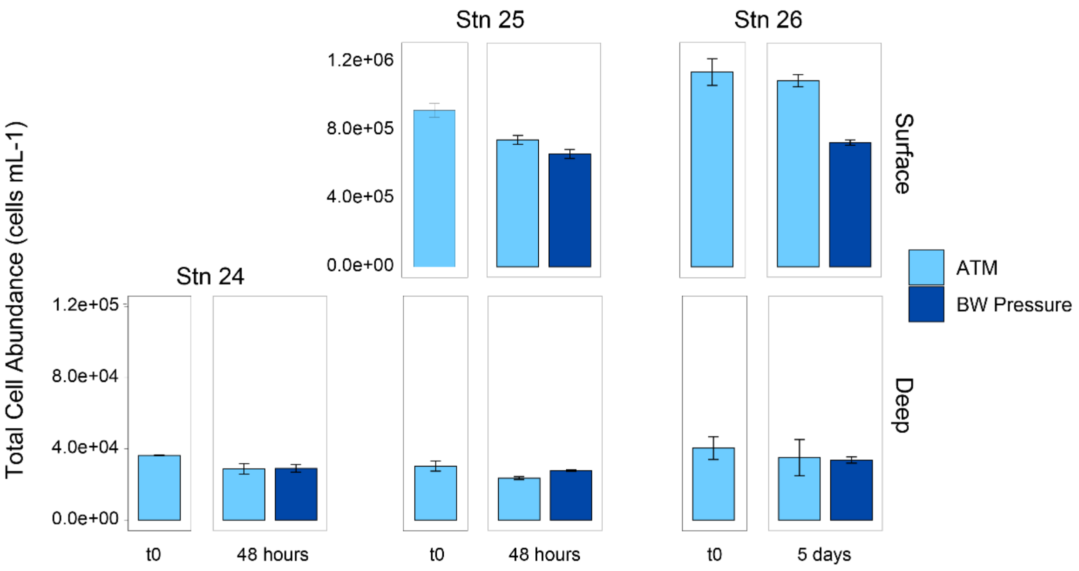

Figure S5. Total cell abundance from surface and deep open-ocean waters at three stations in the North Atlantic Ocean. Samples were incubated under atmospheric pressure or in situ pressures equivalent to deep water sampling (i.e., bottom water pressure) at each station. Bars represent the average of triplicate incubations while error bars represent the standard deviation of these incubations. Note differences in the y-axes between depths.

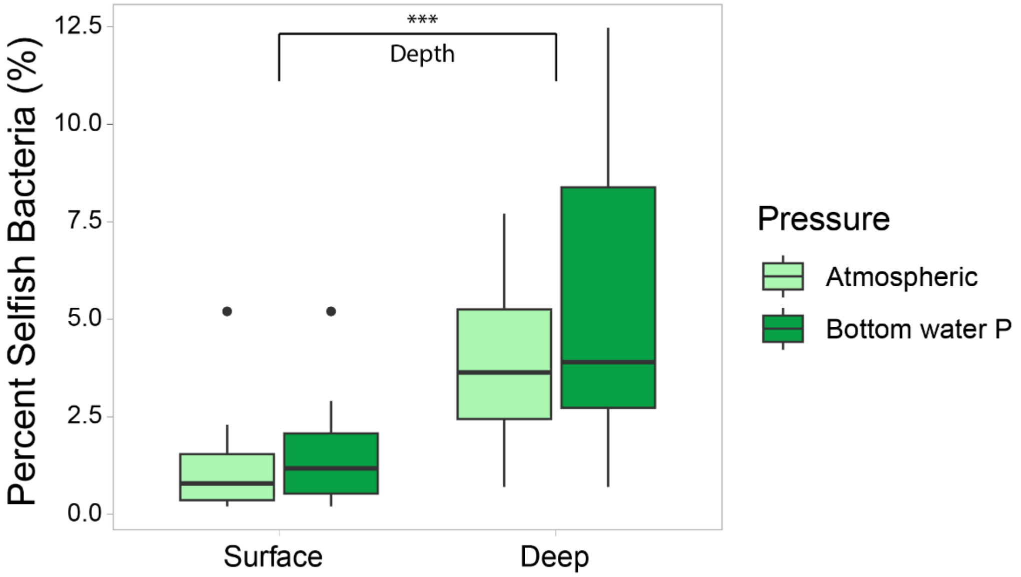

Figure S6. The percentage of selfish bacteria in the surface and deep ocean at all stations. Light green shows incubations at atmospheric pressure, dark green shows incubations that were re-pressurized to bottom water pressure at each station. The box represents the upper and lower quartiles of the dataset while the line represents the median; whiskers represent the standard deviation, and dots are outliers.

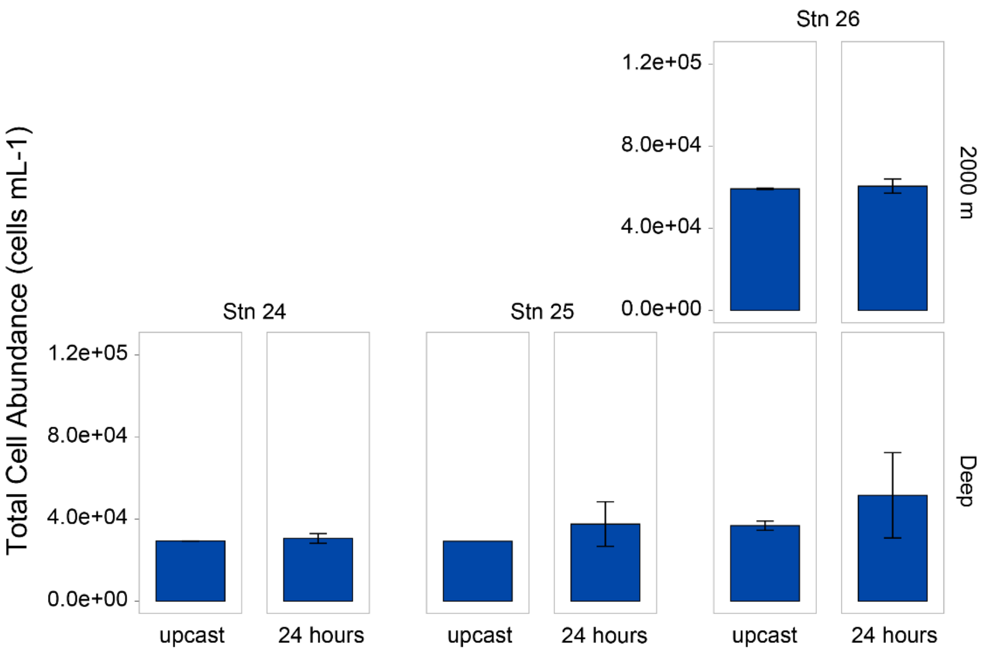

Figure S7. Total bacteria abundance of samples collected from in-situ syringe system deployments at either 2000 m or bottom water (deep ocean). Bars represent the average of triplicate incubations while error bars represent the standard deviation of these incubations. ‘upcast’ represents samples collected from syringes that were triggered 5 minutes prior to retrieval of the syringe system, while the other samples were incubated for 24 hours at in situ temperature and pressure prior to retrieval of the syringe system.
